# The Impact of Granulocyte Colony-Stimulating Factor Administration in a Neutropenic Cancer Patient With COVID-19-Related Acute Respiratory Distress Syndrome

**DOI:** 10.7759/cureus.35399

**Published:** 2023-02-24

**Authors:** Ayman Mohamed, Shirin Zavoshi, Rabia Mahmood, Harish Gidda

**Affiliations:** 1 Internal Medicine, Ascension St. John Hospital, Detroit, USA

**Keywords:** febrile neutropenia, neutrophil to lymphocyte ratio (nlr), granulocyte colony-stimulating factor, covid 19, acute respiratory distress syndrome [ards]

## Abstract

Chemotherapy-induced neutropenia is a serious adverse effect found in cancer patients treated with chemotherapy. As these patients are at risk of infections, granulocyte colony-stimulating factors (G-CSF) are commonly used in these patients to increase neutrophil counts. This report describes a case of a 73-year-old female with metastatic breast cancer treated with letrozole and palbociclib who presented to the hospital with flu-like symptoms and a positive SARS-CoV-2 test. She was saturating well on room air without the need for supplemental oxygen initially, however, she was febrile and lab work revealed neutropenia. Subsequently, she was given two doses of Tbo-filgrastim. Her respiratory status deteriorated shortly afterward and she required supplemental oxygen. The chest X-ray obtained at that time revealed increased atelectasis or infiltration in the middle and lower lung fields, and computed tomography angiography of the chest revealed bilateral patchy airspace and ground glass opacities. The timeline from symptom onset along with her imaging findings suggested COVID-19-related acute respiratory distress syndrome (ARDS) as a possible explanation for her respiratory status decline. Interestingly, her neutrophil-to-lymphocyte ratio (NLR) had consistently increased, along with her respiratory status deterioration, after the completion of the two doses of G-CSF. The patient was treated with dexamethasone. Her respiratory status eventually improved prior to discharge.

## Introduction

Acute respiratory distress syndrome (ARDS) caused by the severe acute respiratory syndrome coronavirus 2 (SARS-CoV-2) displays distinctive characteristics that set it apart from ARDS caused by other factors. The onset of ARDS related to COVID-19 often occurs outside the seven-day period established by the Berlin criteria for ARDS diagnosis [1]. Moreover, COVID-19-related ARDS may exhibit normal or elevated lung compliance, contrasting with the reduced lung compliance frequently observed in ARDS caused by other factors [1]. The treatment of neutropenic cancer patients undergoing chemotherapy, who are also found to be infected with COVID-19, presents a significant challenge as it requires a delicate balance between benefits and risks. The use of granulocyte colony-stimulating factors (G-CSF) in neutropenic cancer patients with fever has been well documented for their role in promoting the proliferation and differentiation of neutrophil cells [2,3]. Despite their widely acknowledged therapeutic benefits, there have been several reports in the literature suggesting that their use in COVID-19-positive patients may be associated with worse clinical outcomes, particularly with the increase in the neutrophil-to-lymphocyte ratio [4,5]. This case report presents the case of a 73-year-old cancer patient who was also COVID-19 positive and presented with fever and neutropenia. The patient was treated with two doses of G-CSF. She then experienced a sudden decline in respiratory status soon after, with findings suggesting COVID-19-related ARDS, along with an increased neutrophil-to-lymphocyte ratio.

## Case presentation

A 73-year-old female patient with a medical history significant for metastatic breast cancer treated with palbociclib and letrozole, as well as hypertension, presented to our medical facility with a chief complaint of viral-like symptoms, including fever, chills, nausea, poor appetite, and generalized weakness. Her symptoms started six days prior to her admission. The patient did not report any symptoms of shortness of breath, sore throat, or chest pain. She was unvaccinated against COVID-19. Her last cycle of palbociclib was completed one week prior to presentation.

**Figure 1:**
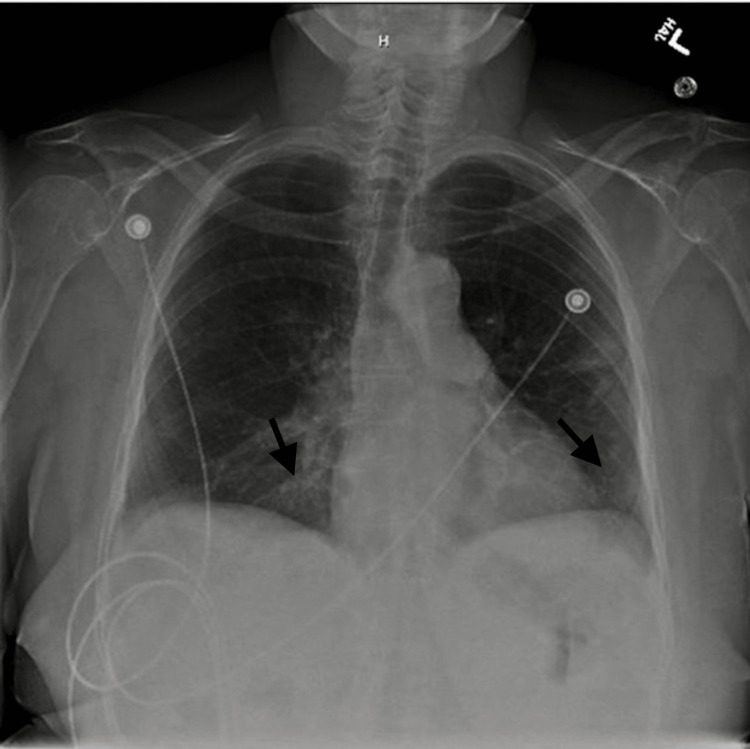
A chest radiograph showing mild infiltrates in the left lower lung and right lung base (arrow)

Upon presentation, the patient exhibited a fever of 101.2 degrees Fahrenheit, blood pressure of 102/54 mmHg, heart rate of 88 beats per minute, respiratory rate of 18 breaths per minute, and oxygen saturation of 98% on room air. Physical examination was normal. Laboratory results were significant for a white blood cell count of 0.59 K/mcL with a neutrophil count of 0.25 K/mcL. A COVID-19 polymerase chain reaction test was positive. D-dimer, lactate dehydrogenase (LDH), erythrocyte sedimentation rate (ESR), ferritin, and C-reactive protein (CRP) were elevated. A chest radiograph was obtained, which revealed mild infiltrates in the left lower lung and right lung base (Figure 1).

Given the patient's fever and neutropenia, it was decided to discontinue palbociclib while in the hospital and to start cefepime. She was also administered two doses of Tbo-filgrastim at 300 mcg on Days 1 and 2 of admission, resulting in the resolution of the neutropenia after two treatments. 

**Figure 2:**
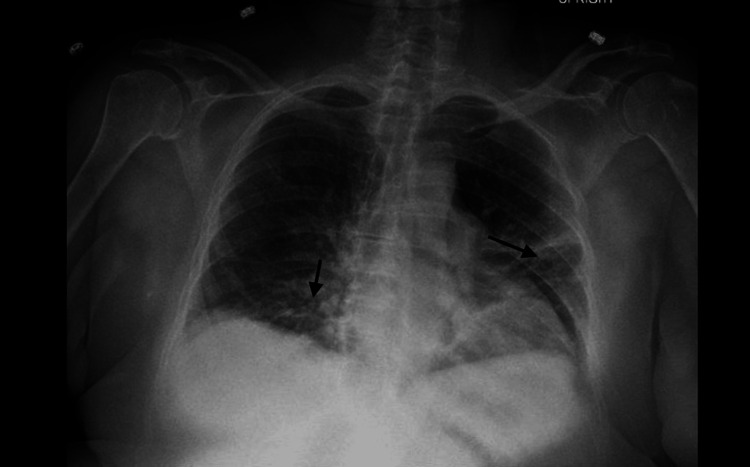
Chest radiograph showing an increase in atelectasis or infiltration within the middle and lower lung regions (arrows)

**Figure 3:**
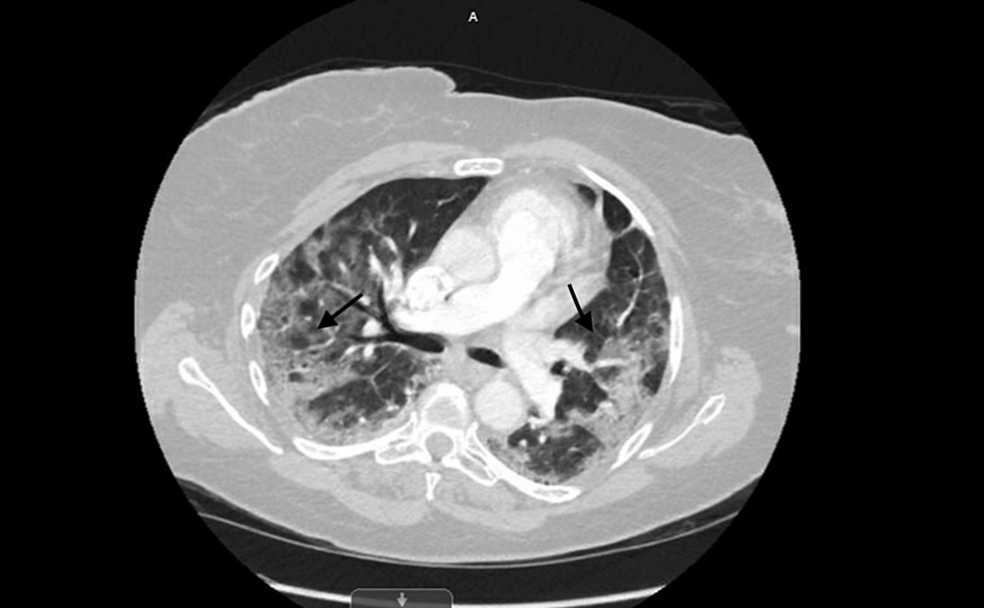
Computed tomography angiography of the chest showing bilateral patchy and ground-glass opacities (arrows).

On the third day of hospital admission, and Day 9 from symptom onset, an increase in the patient's absolute neutrophil count was noted, rising from a baseline value of 0.25 K/mcL to 2.41 K/mcL. The patient's respiratory status then started to decline, along with a decrease in the patient's oxygen saturation levels, which reached a low point of 89% on room air on the same day, but improved to 93% when a 3-liter nasal cannula was employed. Subsequently, a chest radiograph was performed, revealing evidence of atelectasis or infiltration in the middle and lower lung regions (Figure 2). To rule out the possibility of pulmonary embolism, a computed tomography angiography of the chest was also carried out, revealing bilateral patchy airspace and ground-glass opacities, suggesting ARDS (Figure 3). An arterial blood gas analysis was performed and revealed a PaO2 level of 61 mmHg.

Her respiratory status decline was attributed to worsening COVID-19 infection and she was started on a course of dexamethasone 6 mg orally once daily. Over the next seven days, her respiratory status worsened, requiring the use of a high-flow nasal cannula with increasing volumes of oxygen up to 15 L/min. The dose of dexamethasone was increased to twice daily. Over the following six days, the patient's respiratory status showed signs of improvement, and upon discharge, she was only requiring 2 liters of oxygen via nasal cannula. On follow-up, the patient was saturating well on room air four days after discharge.

## Discussion

According to the Berlin criteria, the diagnosis of ARDS is established based on the following: an acute deterioration in respiratory function accompanied by hypoxemia, occurring within one week of a known inciting event, along with evidence of bilateral opacities on chest radiography or computed tomography scans, in the absence of cardiac involvement. These criteria serve as a standard for the diagnosis of ARDS. The onset of ARDS is one of the differences between COVID-19-related ARDS and those resulting from other causes. Studies have indicated that the onset of ARDS in COVID-19 patients occurs between 8-14 days of symptom onset [1]. Our patient exhibited clinical deterioration on the ninth day following symptom onset, possibly as a consequence of the pathophysiological mechanism of ARDS triggered by SARS-CoV-2 infection, which aligns with the expected temporal pattern of COVID-19-related ARDS. However, the potential impact of G-CSF administration on the patient's clinical status is of interest and merits further investigation.

The initiation of G-CSF treatment during the first and second days of the patient's hospital admission led to an improvement in the neutrophil count by the third day of admission. Nevertheless, a simultaneous decline in the patient's clinical state was noted, requiring an increase in oxygen support. The effects of G-CSF administration were investigated in an observational cohort study that enrolled 379 actively treated cancer patients with a confirmed diagnosis of COVID-19. The study aimed to assess the relationship between the degree of neutropenia and the therapeutic response to G-CSF in COVID-19-positive patients, as well as to evaluate the rates of hospitalization, increased oxygen requirements, and mortality [6]. The study found that in the outpatient setting, patients who received G-CSF factor had an increased risk of hospitalization, with a hazard ratio of 3.54 [6]. Additionally, it was observed that in-hospital patients that received G-CSF and had a high response to G-CSF therapy, defined as >50 percentile increase in absolute neutrophil count, had increased levels of oxygen supplementation requirements and even death compared to those with a low response to G-CSF, with a hazard ratio of 3.56. The study concluded that the administration of G-CSF to cancer patients who are neutropenic and COVID-positive should be approached with caution [6].

The neutrophil-to-lymphocyte ratio (NLR) has been demonstrated to possess predictive value for the prognosis of COVID-19 infections, as evidenced by multiple studies in the literature [4,7]. A prospective cohort study that evaluated 352 patients with confirmed COVID-19 infections found that the NLR was higher in patients who experienced clinical deterioration, with a median value of 5.33 compared to 2.14 in patients who did not experience deterioration [5]. Additionally, the NLR was found to be higher in patients who experienced serious clinical outcomes such as shock and death, with a median value of 6.19 in patients who experienced shock and a median value of 7.19 associated with death [6]. Another retrospective study, which evaluated 370 patients with COVID-19 admitted to the intensive care unit, reported a lethality rate of 39.3%. Along with this, cases that resulted in death were found to have an elevated NLR of 7.83 in comparison to survivors who had an NLR of 2.58 [8].

Although our patient respiratory status deterioration was attributed to her COVID-19 infection, her NLR showed an interesting trend. She initially demonstrated adequate saturation on room air on presentation, with a neutrophil-lymphocyte ratio (NLR) within the range of 1.5-2.5. However, after receiving two doses of G-CSF, there was an observable increase in the NLR to 5.5 on Day 3 of admission, which correlated with her respiratory status decline. Subsequently, the NLR continued to rise, reaching a ratio of 9.2 on the tenth day of admission, at which point the patient required 15 L/min of oxygen. The observed increase in NLR was found to be positively correlated with both a decline in the patient's respiratory status and the presence of worsening infiltrative changes on chest radiography.

## Conclusions

Distinguishing COVID-19-related ARDS from ARDS caused by other etiologies is critical in guiding appropriate treatment measures. Given that the timeline from symptom onset is a distinguishing factor, it assumes a significant role in clinical decision-making. In managing neutropenic cancer patients with COVID-19-related ARDS and worsening respiratory function, it is prudent to exercise caution with regards to G-CSF administration. This is due to the potential for G-CSF to result in poorer clinical outcomes.
